# Seroprevalence and Associated Factors of HBV and HCV among Pregnant Women Attending Antenatal Care at Debre Tabor Comprehensive Specialized Hospital, Northwest Ethiopia: A Cross-Sectional Study

**DOI:** 10.1155/2023/2282673

**Published:** 2023-07-31

**Authors:** Ayenew Assefa, Teklehaimanot Kiros, Birtukan Delelegn

**Affiliations:** ^1^Unit of Immunology, Department of Medical Laboratory Science, Debre Tabor University, Debre Tabor, Ethiopia; ^2^Unit of Medical Microbiology, Department of Medical Laboratory Science, Debre Tabor University, Debre Tabor, Ethiopia; ^3^Department of Medical Laboratory Science, Debre Tabor University, Debre Tabor, Ethiopia

## Abstract

**Background:**

Infections with the hepatitis B virus (HBV) and the hepatitis C virus (HCV) are worldwide problems that particularly place a heavy burden on developing nations. HBV and HCV infections during pregnancy have a high rate of vertical transmission and harmful consequences for both the mother and the child. Therefore, this study was carried out to assess the seroprevalence and associated factors of HBV and HCV infections among pregnant women attending antenatal care at Debre Tabor Comprehensive Specialized Hospital in Ethiopia.

**Methods:**

A cross-sectional study was conducted from March 15^th^ to September 16^th^, 2022, at the Debre Tabor Comprehensive Specialized Hospital antenatal care clinic. Five milliliters of venous blood were collected from 422 pregnant women selected using a simple random sampling method. Data on sociodemographic characteristics and risk factors were collected using a prestructured questionnaire. A chi-square test, bivariate, and multivariate analyses were used to evaluate the association between dependent and independent variables. *p* values less than 0.05 were considered statistically significant.

**Results:**

The seroprevalence of HBV and HCV infections was found to be 13% and 0.5%, respectively. Undertaking blood transfusion (AOR = 14.2, CI = 5.81–34.526, *p* = 0.001), tattooing (AOR = 3.99, CI = 1.1–14.36, *p* = 0.034), and dental therapy (AOR = 4.9, CI = 1.41–17.025, *p* = 0.012) were significantly associated with HBV infection.

**Conclusion:**

HBV infection in pregnant women was shown to have a high endemicity (13%) in this investigation, whereas the seroprevalence of HCV infection was low (0.5%). HBV infection was significantly associated with a history of blood transfusions, tattooing, and dental therapy. Screening pregnant women for HBV and HCV infections and providing effective therapy would ensure better outcomes for the newborn. In addition, health education must be used to increase knowledge of screening and modes of transmission.

## 1. Introduction

Globally, viral hepatitis is a significant public health burden. It is thought to have caused 1.4 million deaths, which is more than HIV's 1.2 million death toll [1]. The most widespread viral hepatitis globally is caused by the hepatitis B virus (HBV) and the hepatitis C virus (HCV). HBV has a circular genome of partially double-stranded DNA, while HCV is a single-stranded RNA virus [2, 3]. Chronic HBV and HCV infections affect 248 million and 150 million persons worldwide, respectively, resulting in 780,000 and 350,000 fatalities each year [4].

Vertical transmission, contact of nonintact skin or mucous membranes with secreting or saliva-containing individuals within households, unsafe sexual contact, transfusion of HBV-infected blood and blood products, nosocomial infection, and percutaneous inoculation are all ways that HBV can spread, while perinatal and early childhood transmissions are the primary methods of infection in endemic locations [5, 6]. Direct percutaneous inoculation is the method of HCV transmission that is most effective. Numerous studies have shown that HCV infections in the home, workplace, sexual relationships, and vertically are all significant risk factors [7].

Although they frequently have no symptoms, chronic HBV and HCV infections can cause liver cirrhosis and hepatocellular carcinoma. Therefore, the majority of infected individuals are not aware of their HBV or HCV status until serious liver damage has taken place [8]. Both HBV and HCV cause acute and chronic liver damage, with a considerable mortality rate in mother-child pairs [9]. A higher risk of problems during pregnancy exists in women who have hepatitis [10]. Premature membrane rupture, placenta previa, preterm birth, placental separation, vaginal bleeding, preterm labour, gestational diabetes mellitus, and mortality are among the significant risks of maternal problems associated with viral hepatitis during pregnancy [11].

Sub-Saharan Africa has a high prevalence of hepatitis B and C virus infections [3]. Hepatitis B surface antigen (HBsAg) is typically used as the foundation for a laboratory diagnosis of HBV infection. The prevalence of HBsAg carriers varies between 3% and 20% in sub-Saharan Africa, depending on the region, risk categories, and age of the population [12]. In Africa, the prevalence of HCV in the general population ranges from 0.1% to 17.5%, depending on the nation [13]. According to a study done in Addis Ababa, Ethiopia, the average prevalence of HBsAg was 6.1%, and the overall prevalence of HCV among the population was 0.9% and 1.3% among individuals over the age of 15 [14]. There are limitations to regional studies in African countries on the seroprevalence of HBV and HCV in pregnant women, where the majority of epidemiological studies were carried out on individuals among specific groups, such as blood donors, healthcare workers, or hemodialysis patients. As a result, the purpose of this study was to determine the seroprevalence and associated factors of HBV and HCV among pregnant women receiving antenatal care at Debre Tabor Comprehensive Specialized Hospital in North West Ethiopia.

## 2. Methods and Materials

### 2.1. Study Area, Design, and Period

The study was conducted at Debre Tabor Comprehensive Specialized Hospital in the South Gondar Zone of the Amhara Regional State, which is located 654 km far from the capital city, Addis Ababa. There is one public hospital, three public health centers, four public health posts, and three private clinics in the town. The hospital provides health services for over 2.7 million inhabitants in the town and surrounding areas. A cross-sectional study was conducted among pregnant women attending ANC at Debre Tabor Comprehensive Specialized Hospital from March 15^th^ to September 16^th^, 2022.

### 2.2. Source and Study Population

The source population was all pregnant women visiting ANC at Debre Tabor Comprehensive Specialized Hospital, while all pregnant women who accepted informed consent during the data collection period were the study subjects.

### 2.3. Inclusion and Exclusion Criteria

Pregnant women who were willing to participate and provide consent were included in the study, whereas pregnant women who attended ANC but were unable to communicate due to serious illness were excluded.

### 2.4. Study Variables

The dependent variables were seropositivity for HBV and HCV, while maternal age, religion, residence, occupational status, educational status, gestational age, gravida, history of surgical procedures, body tattooing, history of blood transfusion, and dental therapy were the independent variables.

### 2.5. Sample Size Determination and Sampling Technique

The simple random sampling technique was applied depending on the patient's random accessibility. The sample size was computed using 50% prevalence to achieve the maximum sample size, and a single population proportion formula *N* = (*Z*_*a*/2_)^2^ × *p* (1−*p*)/*D*^2^. Based on the following assumptions: the 0.05 significance level, *Z*_*a*/2_ = 95% confidence interval = 1.96, margin of sampling error = 0.05, and a 10% nonresponse rate, we arrive at a sample size of 422.

### 2.6. Data Collection

#### 2.6.1. Sociodemographic Characteristics and Clinical Factors

After obtaining informed consent, a structured questionnaire was given to eligible pregnant women for an interview in order to gather sociodemographic data, including information on the mother's age, religion, occupation, place of residence, marital status, level of education, and other details on potential risk factors for the transmission of HBV and HCV, such as gestational age, gravida, history of blood transfusions, dental therapy, history of surgical procedures, and tattooing.

#### 2.6.2. Specimen Collection and Processing

Five milliliters of venous blood were collected by laboratory technologists and left for 30 minutes to aid in clotting. Following that, the blood samples were centrifuged for five minutes at room temperature at a speed of 3000 revolutions per minute. The leftover serum was separated, collected, kept at room temperature, and subjected to a rapid strip test to determine HBsAg and anti-HCV. The one-step HBsAg rapid test kit with sensitivity and specificity of 95.5% and 98.6%, respectively (Nantong Egens Biotechnology Co., Ltd., China) was used following the manufacturer's instructions to detect HBsAg. HCV rapid test strip (Assure Tech. (Hangzhou) Co., Ltd., China) with 100% sensitivity and 99.4% specificity was used in accordance with the manufacturer's instructions to detect anti-HCV antibodies.

### 2.7. Data Analysis

Data entry and data analysis were carried out using SPSS version 20 software. To determine the presence and degree of association between the independent and outcome variables, the chi-square test, bivariate, and multivariate analyses were utilized. All variables with a *p* value less than 0.25 in bivariate analysis were selected for multivariate analysis. The proportion of positive pregnant women in the entire study population taken into account was used to calculate the prevalence of HBV and HCV, which was then reported as a percentage. Hosmer and Lemeshow statistics were used to determine the model's fitness. *P* values less than 0.05 were considered significant with 95% confidence intervals.

### 2.8. Quality Assurance

The questionnaire was initially written in English, then translated into Amharic, and finally returned to English. This was carried out to preserve simplicity, completeness, and clarity. Prior to the study, 5% of the questionnaire was pretested. It was evaluated on how long it took to complete the questionnaire and how each question flowed and was clear and easy to understand. The principal investigator regularly verified the accuracy of all the data collected.

### 2.9. Ethical Consideration

This study was carried out after getting ethical clearance and permission from the institutional research committee (IRC) of Debre Tabor University. A letter of support was written to Debre Tabor Comprehensive Specialized Hospital. Information about the study was given to all pregnant women, and they were assured of the confidentiality, protection, and anonymity of their data. Verbal informed consent was obtained from the study participants before data collection.

## 3. Results

### 3.1. Sociodemographic and Clinical Data of Study Subjects

A total of 422 pregnant women were included in the study. The majority (38.2%) of pregnant women were in the age group of 25–29. The mean age of the study participants was 25.87 ± 1.452 SD. The minimum and maximum ages were 17 and 41 years, respectively. Of the total pregnant mothers included in this study, 252 (59.7%) were urban dwellers (Table 1).

### 3.2. Seroprevalence of HBsAg and Anti-HCV Antibody

Fifty-five (13%) and two (0.5%) of the participants tested positive for HBsAg and anti-HCV, respectively. None of the pregnant women had both HBV and HCV infections (Table 2).

### 3.3. Relationship between HBV and HCV Seropositivity with Sociodemographic Characteristics

The majority (41.8%) of HBV-positive pregnant women were in the age group of 25–29. There was a statistically significant association between the age categories and the distribution of HBsAg (*p*=0.012). All pregnant women who tested positive for HCV and 90.9% of those who tested positive for HBV were married. The marital status of the participants and HBsAg were statistically significantly associated (*p*=0.009). The prevalence of HBsAg positivity was 52.7% in urban residents and 47.3% in rural dwellers. However, there was no statistically significant association found between residence and seroprevalence of HBsAg (*p*=0.26). Most (25.5%) of pregnant women who tested positive for HBV could only read and write, while all of those who tested positive for HCV had attended secondary school. No statistically significant association exists between HBsAg distribution and level of education (*p*=0.29) (Table 3).

Among occupations, farmers were found to have the highest (40.0%) frequency of HBV infection, followed by merchants (23.6%) and housewives (23.6%). No statistically significant association between HBsAg positivity and occupation was found in this study (*p*=0.544). When comparing primigravida and multigravida women, the seroprevalence of HBsAg was 13.8% for the former and 12.3% for the latter. The distribution of HBsAg and the study subjects' gravida, however, showed no statistically significant relationship (*p*=0.65). For the first, second, and third trimesters, the prevalence of HBV was 8.55%, 15.7%, and 15.3%, respectively. However, there was no statistically significant association between the prevalence of HBsAg and gestational age (*p*=0.12).

### 3.4. Factors Associated with HBV

About twelve percent (11.8%) of pregnant women had a history of receiving blood transfusions, and 52% of them tested positive for HBsAg. A statistically significant association between the history of blood transfusion and HBV infection was found (AOR = 14.2, 95% CI = 5.81–34.52, and *p*=0.001). The percentage of study participants who had a history of tattoos was 12.8%, of which 42.6% were positive for HBsAg. There was a statistically significant association between HBV infection and tattoo history (AOR = 3.99, 95% CI = 1.1–14.36, and *p*=0.034). Of the 63 pregnant women who had a history of dental therapy, 41.3% tested positive for HBsAg. A statistically significant association between these variables was found (AOR = 4.9, 95% CI = 1.412–17.025, and *p*=0.012) (Table 4).

## 4. Discussions

Both HBV and HCV infections are infectious illnesses that can be spread horizontally by body fluids and blood products or vertically from mothers to their newborns [15]. The prevalence of HBV and HCV infections among the participants in the present study was 13% and 0.5%, respectively. According to WHO standards for the global epidemiology of HBV infection, the 13% prevalence of HBsAg found in the current study area appears to be high endemicity [16]. The anti-HCV antibody seroprevalence is in line with the WHO low criterion [17]. Our investigation did not uncover any evidence of coinfection with HBV or HCV, which is consistent with the findings of Murad et al. [18] and a Bahir Dar study [8]. This may be attributed to the low prevalence of HCV infection in the present study.

The seroprevalence of HBsAg in this study was higher than that found in studies conducted in other regions of Ethiopia, including those at Borumeda General Hospital (8.1%) [19], Dilla University Referral Hospital (5.1%) [20], Gedeo Zone (9.2%) [21], Bahirdar (3.8%) [22], and Addis Ababa (6%) [23]. Moreover, the current result is higher than the findings of similar studies from Yemen 10.8% [18], Tanzania 8.03% [24], Ghana 7.7% [25], and the Democratic Republic of the Congo 3.9% [26]. This diversity could be brought on by the type of population investigated, distinct geographic locations, genetic variables, socioeconomic position, regional variations in risk factors, and cultural behaviors. The variance may also result from variations in the study population's sample size, participant knowledge of HBV transmission routes, and safety measures.

HCV prevalence was comparable to studies from Spain [27], India [28], Tanzania [29], and Bahirdar [8] that found 0.26%, 0.21%, 0.3%, and 0.26%, respectively. Nevertheless, it was lower than the results in East Wollega (8.07%) [30]; in Yemen (8.5%) [18]; in Western Ethiopia (8.1%) [31]; in Iran (1.33%) [32]; and in Nigeria (2.7%) [33]. These inconsistencies may be explained by variations in the study population's behavior, cultural customs, the diagnostic tools utilized, and demographics related to the risk of HCV infection.

According to our research, participants in the study who had a history of blood transfusions were 14.2 times more likely to be HBsAg seropositive. A related study carried out at the Sankura Primary Hospital in southern Ethiopia showed that the risk of HBV infection increased by 4.8 times [34]. Another study conducted in Ethiopia also showed that pregnant women with a history of transfusions are 5.71 times more likely to contract HBV [35]. The preseroconversion window period and occult HBV infection, which are marked by the absence of measurable HBsAg, appear to be connected with transfusion transmission. Therefore, it is conceivable to utilize additional HBV markers and HBV nucleic acid tests in HBV screening [36]. A history of blood transfusions, however, was not found to be substantially associated with the presence of HBsAg in a study carried out at Yirgalem Hospital in Ethiopia [37]. This discrepancy might be caused by the difference in sample sizes as well as differences in study design, target population, and study settings.

A known risk factor for HBV infection is tattooing, which is carried out as a kind of body modification for beauty and aesthetic appeal [38]. Participants in this study who had a previous history of tattooing were 4 times more likely to test positive for HBsAg. This result is in line with a study done at Debre Markos Referral Hospital, which found that body tattooing practice increased the likelihood of testing positive for HBsAg by 4.94 times [39], and a study conducted in the town of Ambo, in the regional state of Oromia, Ethiopia, found that a history of practicing tattoos increased HBsAg positivity risk by 5.31 times [40].

In this study, the risk of HBV infection in pregnant women who had received dental care increased by 4.9 times. This outcome was in line with research carried out in Pakistan [41], as well as in a study by Qureshi et al. [42]. Nevertheless, studies by Jagannathan et al. in India [43], and Ozer et al. in Turkey [44], found no association between dental therapy and HBV infection. The risk of dental therapy for HBV infection is related to things like poor sterilizing practices, unsafe injections, treatments given by unqualified personnel, and the lack of use of protective equipment [45].

## 5. Conclusion and Recommendations

In this study, the seroprevalence of HBV and HCV infection was 13% and 0.5%, respectively. HBV infection was significantly associated with a history of blood transfusions, tattooing, and dental therapy. Better outcomes for the baby are guaranteed when maternal hepatitis is properly managed during the prenatal stage, and health education about modes of transmission and screening should be introduced during ANC follow-up. In addition to the rapid tests, more sensitive assay methods like ELISA and polymerase chain reaction (PCR) should be included in future studies to screen for HBV and HCV infections.

## Figures and Tables

**Table 1:** Sociodemographic characteristics and clinical information of study participants at Debre Tabor Comprehensive Specialized Hospital, March 15^th^ to September 16^th^, 2022.

Variables	Level	Frequency (*N*)	Percent (%)
Age in years	15–19	39	9.2
	20–24	128	30.3
	25–29	161	38.2
	30–34	72	17.1
	35–39	21	5.0
	40–44	1	0.2

Residence	Urban	252	59.7
	Rural	170	40.3

Religion	Orthodox	376	89.1
	Muslim	46	10.9

Marital status	Married	406	96.2
	Single	1	0.2
	Divorced	15	3.6

Educational status	Unable to read and write	64	15.2
	Only read and write	82	19.4
	Primary school (1–8)	72	17.1
	Secondary school (9-10)	38	9.0
	Preparatory (11-12)	67	15.9
	College and above	99	23.5

Occupational status	Governmental employee	57	13.5
	Housewife	86	20.4
	Merchant	112	26.5
	Farmer	152	36.0
	Other	15	3.6

Gestational age	First trimester	152	36.0
	Second trimester	172	40.8
	Third trimester	98	23.2

Gravida	Prim gravida	203	48.1
	Multigravida	219	51.9

History of blood transfusion	Yes	372	88.2
	No	50	11.8

History of surgery	Yes	387	91.7
	No	35	8.3

History of tattoo	Yes	368	87.2
	No	54	12.8

History of dental therapy	Yes	359	85.1
	No	63	14.9

**Table 2:** Seroprevalence of HBV and HCV infections among pregnant women.

Variables	Status	Total (%)
HBsAg status	Negative	367 (87.0)
	Positive	55 (13.0)

Anti-HCV antibody status	Positive	2 (0.5)
	Negative	420 (99.5)

Coinfection	Positive	0 (0)
	Negative	422 (100)

**Table 3:** HBV and HCV seroprevalence in relation to sociodemographic characteristics.

Variables	Category	*HBV status*			*HCV status*		
		Pos, *N*(%)	Neg, *N*(%)	*p* value	Pos *N*(%)	Neg *N*(%)	*p* value
Age category	15–19	1 (1.8)	38 (10.4)	0.012	0 (0)	39 (9.3)	0.763
	20–24	13 (23.6)	115 (31.3)		1 (50)	127 (30.2)	
	25–29	23 (41.8)	138 (37.6)		0 (0)	161 (38.3)	
	30–34	12 (21.8)	60 (16.3)		1 (50%)	71 (16.9)	
	35–39	5 (9.1)	16 (4.4)		0 (0)	21 (5.0)	
	40–44	1 (1.8)	0 (0)		0 (0)	1 (0.2)	

Religion	Orthodox	46 (83.6)	330 (89.9)	0.163	2 (100%)	374 (89.0)	0.62
	Muslim	9 (16.4)	37 (10.1)		0 (0)	46 (11.0)	

Residence	Urban	29 (52.7)	223 (60.8)	0.26	0 (0)	252 (60)	0.084
	Rural	26 (47.3)	144 (39.2)		2 (100)	168 (40)	

Marital status	Married	50 (90.9)	356 (97.0)	0.009	2 (100)	404 (96.2)	0.96
	Single	1 (1.8)	0 (0)		0 (0)	1 (0.2)	
	Divorced	4 (7.3)	11 (3.0)		0 (0)	15 (3.6)	

Educational status	Unable to read and write	11 (20.0)	53 (14.4)	0.292	0 (0)	64 (15.2)	0.001
	Only read and write	14 (25.5)	68 (18.5)		0 (0)	82 (19.5)	
	Primary school	5 (9.1)	67 (18.3)		0 (0)	72 (17.1)	
	Secondary school	4 (7.3%)	34 (9.3)		2 (100)	36 (8.6)	
	Preparatory	11 (20.0)	56 (15.3)		0 (0)	67 (16.0)	
	College and above	10 (18.2)	89 (24.3)		0 (0)	99 (23.6)	

Occupational status	Governmental employee	7 (12.7)	50 (13.6)	0.544	0 (0)	57 (13.6)	0.865
	Housewife	13 (23.6)	73 (19.9)		0 (0)	86 (20.5)	
	Merchant	13 (23.6)	99 (27.0)		1 (50)	111 (26.4)	
	Farmer	22 (40.0)	130 (35.4)		1 (50)	151 (36.0)	
	Other	0 (0)	15 (4.1)		0 (0)	15 (3.6)	

Gestational age	First trimester	13 (8.6)	139 (91.4)	0.121	1 (50)	151 (36.0)	0.734
	Second trimester	27 (15.7)	145 (84.3)		1 (50)	171 (40.7)	
	Third trimester	15 (15.3)	83 (84.7)		0 (0)	98 (23.3)	

Gravida	Primigravida	28 (13.8)	175 (86.2)	0.655	1 (50)	202 (48.1)	0.96
	Multigravida	27 (12.3)	192 (87.7)		1 (50)	218 (51.9)	

**Table 4:** Bivariate and multivariate analysis of the factors associated with the HBV among pregnant women attending ANC at Debre Tabor Comprehensive Specialized Hospital.

Variables	Category	*HBsAg*		COR (CI)	AOR (CI)	*p* value
		Pos (%)	Neg (%)			
Resident	Urban	29 (6.9)	223 (52.8)	0.72 (0.408–1.279) 1	1.897 (0.885–4.067) 1	0.1
	Rural	26 (6.1)	144 (34.1)			

Marital status	Married	50 (11.8)	356 (34.3)	0.86 (0.118–1.260) 1	2.47 (0.455–13.439) 1	0.295
	Single	1 (0.2%)				
	Divorce	4 (0.9)	11 (2.6)			

Gestational status	First	13 (3.0)	139 (32.9)	1.991 (0.987–4.15) 1	0.791 (0.305–2.051) 1	0.397
	Second	27 (6.3)	145 (34.5)			
	Tertiary	15 (3.5)	83 (19.6)			

History of blood transfusion	Yes	26 (52.0)	24 (48.0)	12.8 (6.54–25.08) 1	14.2 (5.81–34.526) 1	0.001
	No	29 (7.8)	343 (92.2)			

Surgery	Yes	17 (48.6)	18 (51.4)	8.67 (4.13–18.23) 1	0.37 (0.84–1.65) 1	0.193
	No	38 (9.8)	349 (90.2)			

Tattoo	Yes	23 (42.6)	31 (57.4)	7.79 (4.07–14.922) 1	3.99 (1.1–14.36) 1	0.034
	No	32 (8.7)	336 (91.3)			

Dental therapy	Yes	26 (41.3)	37 (58.7)	8 (4.26–15.0) 1	4.9 (1.412–17.025) 1	0.012
	No	29 (8.1)	330 (91.9)			

AOR: adjusted odds ratio; COR: crude odds ratio; CI: confidence interval.

## Data Availability

All data generated or analysed during this study are available from the corresponding author on reasonable request.

## Abbreviations

ANC: Antenatal care
Anti-HCV Ab: Anti-hepatitis C virus antibody
DNA: Deoxyribonucleic acid
ELISA: Enzyme-linked immunosorbent assay
HBV: Hepatitis B virus
HBsAg: Hepatitis B surface antigen
HCV: Hepatitis C virus
RNA: Ribonucleic acid
SPSS: Statistical Package for Social Science
WHO: World Health Organization.
